# Tumor-Infiltrating Lymphocytes and Tumor Budding: Emerging Prognostic Markers in Breast Cancer

**DOI:** 10.7759/cureus.96266

**Published:** 2025-11-06

**Authors:** Naincy Rastogi, Shreshtha Ghosh, Priyanka Sameer, Deepti Mishra, Priyadarshini Guha

**Affiliations:** 1 Department of Pathology, Geetanjali Institute of Medical Sciences, Jaipur, IND; 2 Department of Pathology and Cancer Genetics, Kalyan Singh Super Speciality Cancer Institute, Lucknow, IND; 3 Department of Pathology, Globe Healthcare, Lucknow, IND

**Keywords:** invasive breast cancer, prognostic marker, tumor budding, tumor-infiltrating lymphocytes, tumor microenvironment

## Abstract

Introduction

Invasive breast carcinoma of no special type (IBC-NST) remains a significant global health burden. Tumor-infiltrating lymphocytes (TILs) reflect the host's anti-tumor immunity and are associated with favorable outcomes, whereas tumor budding (TB) is a marker of tumor aggressiveness. This study aimed to assess TILs and TB in IBC-NST and correlate them with clinicopathological parameters.

Methods

This retrospective study included 90 (100%) cases of IBC-NST. Stromal TILs (sTILs) and intratumoral TILs (iTILs) were quantified on hematoxylin and eosin (H&E)-stained sections per international guidelines. Tumor buds were counted at the invasive front and classified into low- and high-grade budding. Associations between TILs, TB, and clinicopathological features were analyzed using chi-squared and correlation tests.

Results

Among 90 cases, 89 (98.8%) were IBC-NST. Both sTILs and iTILs decreased with higher tumor grade, larger size, and increased mitotic count, with statistically significant associations for grade and mitosis (p < 0.05). High-grade TB was significantly associated with lymph node metastasis (35/53, 66.0%), higher tumor grade (50/78, 64.1%), tumor size > 5 cm (23/27, 85.2%), and lymphovascular invasion (32/42, 76.19%) (p < 0.05 for all). A moderate but non-significant positive correlation was noted between sTILs and TB (r = 0.163, p = 0.124).

Conclusion

Higher TIL levels correlate with favorable tumor characteristics, while increased TB reflects aggressive histological features. A combined evaluation of TILs and TB provides valuable prognostic insights and may assist in stratifying breast cancer patients for tailored therapeutic approaches.

## Introduction

Invasive breast cancer remains a significant global health concern, accounting for substantial cancer-related deaths worldwide [1]. Understanding its underlying pathogenesis and identifying potential prognostic factors are crucial for effective treatment. Traditional prognostic factors, including lymph node status, tumor size, histologic type, histologic grade, and lymphovascular invasion (LVI), are well-established. However, in recent years, the significance of tumor microenvironment components, such as tumor budding (TB) and tumor-infiltrating lymphocytes (TILs), has garnered considerable attention for their potential roles in cancer progression and patient outcomes [2]. The complex biology of invasive breast carcinoma can be better understood by elucidating the interrelationships between TB, TILs, and clinicopathological characteristics. Integrating these parameters with established prognostic factors, including tumor grade, histological subtype, hormone receptor status, and lymph node involvement, is essential for uncovering the underlying mechanisms that drive disease progression and treatment response. This integrated approach holds promise for refining prognostication and personalizing therapeutic strategies.

TB, marked by the presence of single or small clusters of dedifferentiated tumor cells at the invasive front, has emerged as a significant histopathological feature with potential prognostic implications. Research in various cancer types has consistently linked increased TB with adverse outcomes, including higher rates of lymph node metastasis, advanced tumor stages, and poorer survival rates [3]. In invasive breast carcinoma, the presence and extent of TB have garnered interest in exploring its correlation with clinicopathological factors and its impact on patient prognosis. The underlying mechanisms of TB involve complex signaling pathways, including TGF-β/SMADs, Notch, and WNT/β-catenin, which regulate the epithelial-to-mesenchymal transition (EMT) process. TB is influenced by the EMT, a process characterized by reduced expression of epithelial markers, such as cytokeratin (CK), along with increased levels of mesenchymal proteins, like vimentin. This transition is also associated with elevated expression of transcription factors, including ZEB, Twist, and Snail, as well as cancer stem cell markers. Furthermore, factors such as hypoxia and pro-inflammatory cytokines in the tumor microenvironment play a pivotal role in regulating this phenomenon.

TILs, comprising diverse immune cell populations, have emerged as crucial components of the tumor microenvironment. The density and composition of TILs are associated with the host's immune response against the tumor and may significantly influence clinical outcomes in breast cancer [4]. Investigating the interplay between TILs and TB in invasive breast carcinoma is essential to elucidate their combined prognostic significance. To standardize TIL evaluation, the International TIL Working Group (ITILWG) published guidelines in 2014, promoting uniformity and objectivity [5]. TILs are broadly classified into two categories: stromal TILs (sTILs), which are located in the stromal tissue and do not directly touch tumor cells, and intratumoral TILs (iTILs), which are situated within the tumor nests and maintain direct contact with the malignant cells.

This study primarily aims to investigate the correlation between TB, TILs, and various clinicopathological features in patients with invasive breast carcinoma. By integrating these components, we seek to identify novel predictive and prognostic biomarkers that can enhance risk stratification and inform treatment decisions. We hypothesize that increased TB and disrupted TIL distribution will be associated with unfavorable clinicopathological factors, indicative of more aggressive tumor behavior and poorer clinical outcomes. The study specifically sought to evaluate the correlation of TILs and TB with key clinicopathological features such as tumor grade, stage, LVI, and mitotic activity.

## Materials and methods

All consecutive cases of invasive breast carcinoma diagnosed between January 2018 and December 2022 that met the inclusion and exclusion criteria were included in the study. Formalin-fixed, paraffin-embedded tissue blocks were sectioned at a uniform thickness of 4 µm and stained with hematoxylin and eosin (H&E) using standard laboratory protocols. TILs were scored according to the ITILWG criteria, assessing 3-5 high-power fields (400×) in areas with the densest infiltration (“hotspots”). TB was evaluated following the International Tumor Budding Consensus Conference (ITBCC) recommendations, selecting a single hotspot at the invasive front and counting buds under 200× magnification.

Two experienced pathologists independently reviewed all slides. Interobserver variability was addressed by consensus review in cases of discrepancy, with the predominant pattern recorded for borderline budding counts or mixed TIL distributions. Representative photomicrographs have been provided, and additional tabulated data illustrate the differences between low and high TB and low and high TILs for standardization and reproducibility across institutions.

This cross-sectional study comprised 90 cases of invasive breast carcinoma, identified from the departmental archives. Cases were selected based on the availability of H&E-stained slides. Modified radical mastectomy specimens and lumpectomies were included, while biopsy cases and carcinoma in situ cases were excluded.

**Figure 1:**
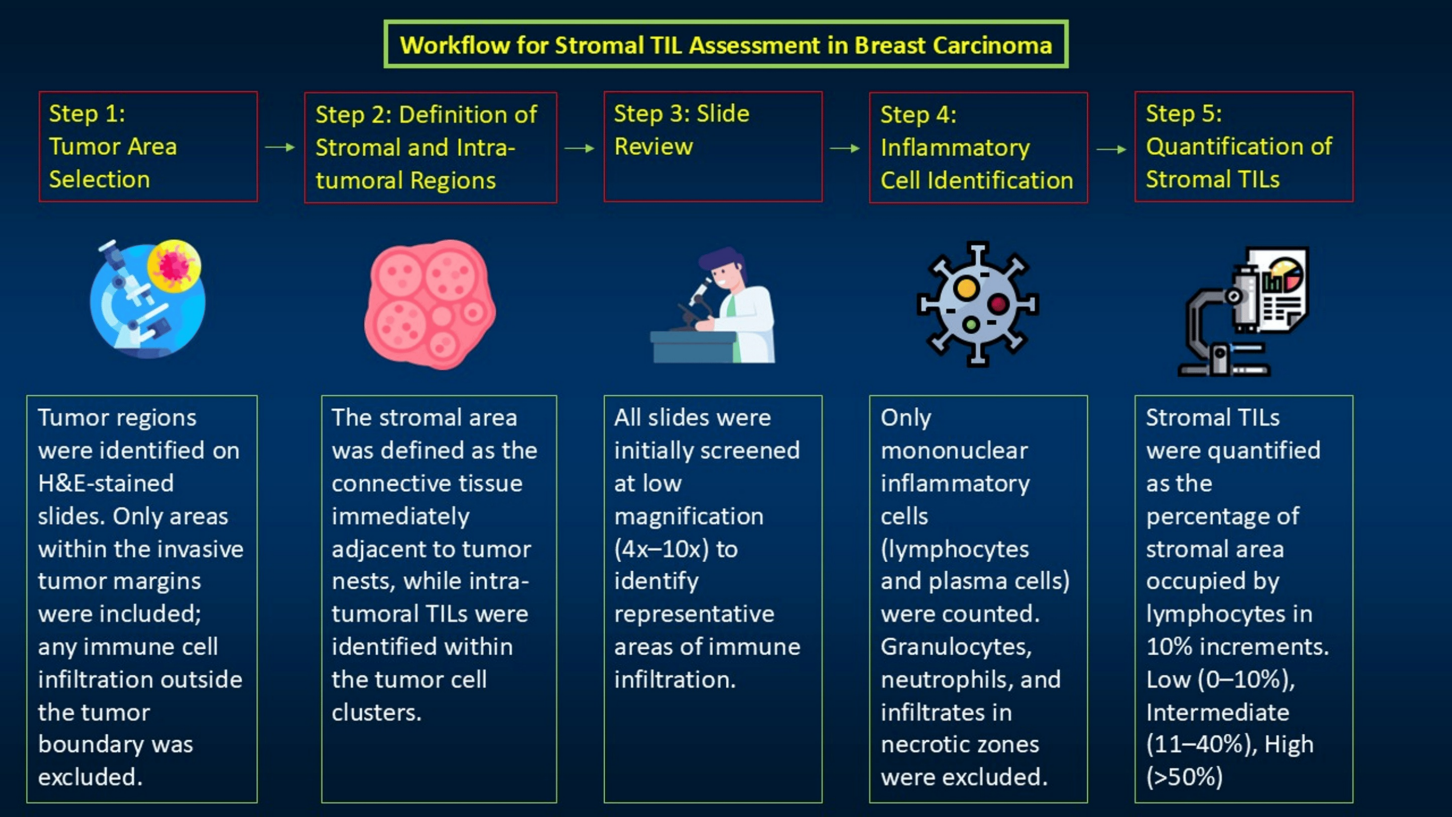
Guidelines for a standardized approach for tumor-infiltrating lymphocyte (TIL) evaluation in breast cancer H&E: hematoxylin and eosin Figure credits: Dr. Naincy Rastogi

For each case, a representative 5 μm H&E-stained section was selected. TB and TILs were assessed by experienced pathologists using standardized criteria. Clinicopathological data, including tumor size, histological grade, lymph node involvement, LVI, and cancer stage, were extracted from patient medical records. TIL assessment was performed according to the ITILWG recommendations (Figure 1).

**Figure 2:**
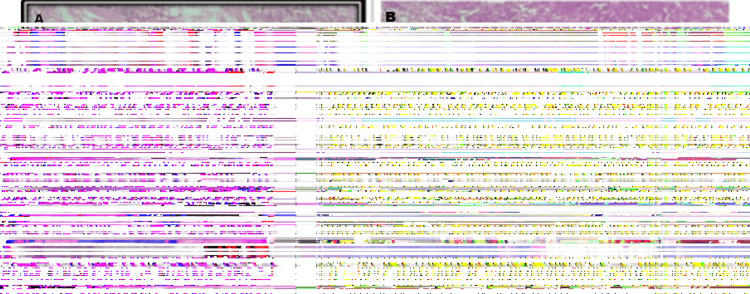
Representative H&E-stained photomicrographs illustrating intratumoral TIL (iTIL) scoring in breast carcinoma cases (A) Score 1: sparsely distributed lymphocytes visible under high magnification; (B) Score 2: moderate infiltration with readily identifiable lymphocytes at intermediate magnification. H&E: hematoxylin and eosin

**Figure 3:**
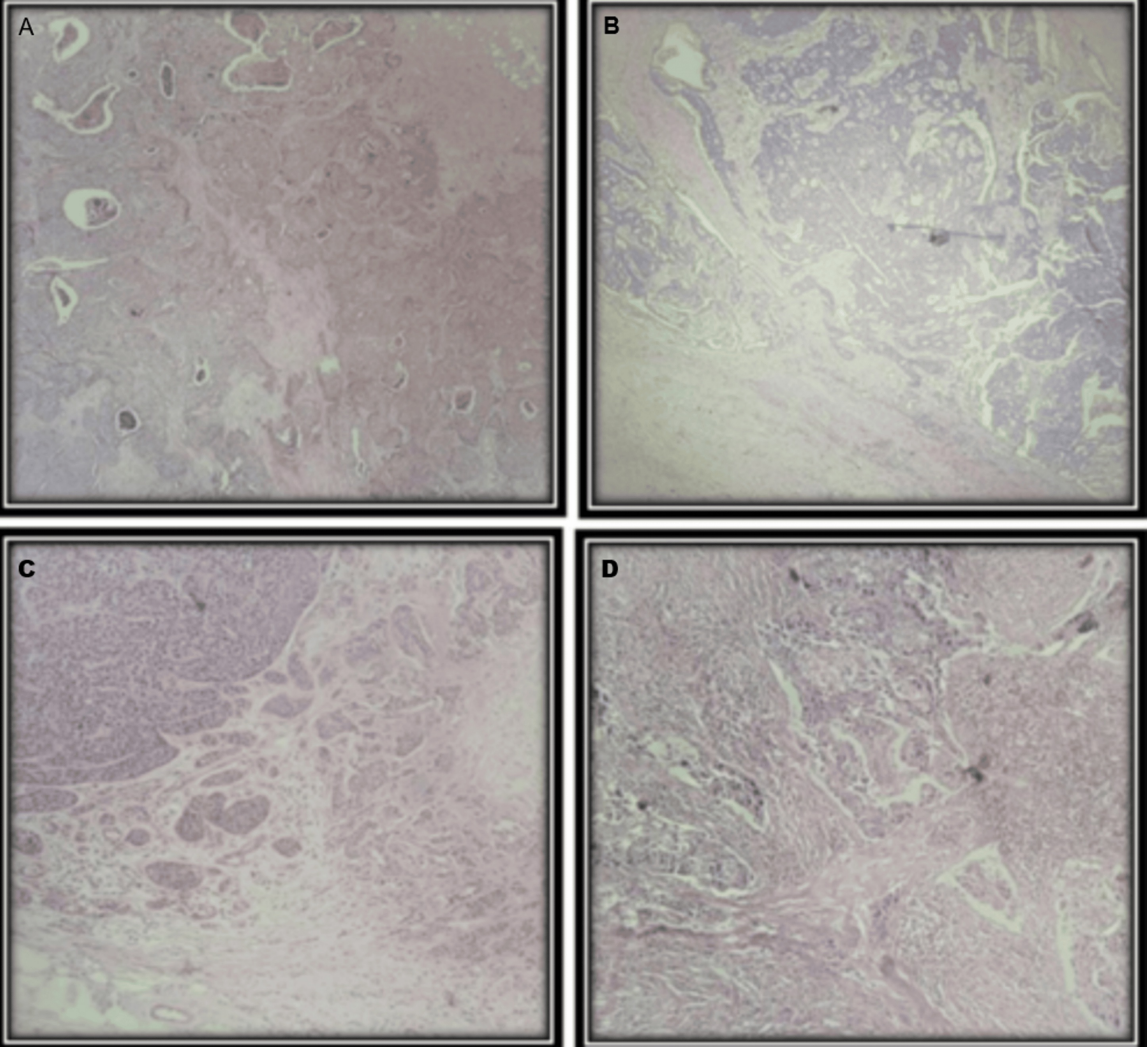
H&E-stained photomicrographs demonstrating stromal TIL (sTIL) evaluation in breast carcinoma cases (A) <10% sTILs, (B) 25%–30% sTILs, (C) 50%–60% sTILs, and (D) 80%–90% sTILs, indicating increasing levels of lymphocytic infiltration within the tumor stroma. H&E: hematoxylin and eosin; TIL: tumor-infiltrating lymphocyte

The percentage of sTILs was evaluated at ×200 magnification within the invasive tumor borders. Lymphoid cells were assessed in the stromal area between tumor nests. sTILs were categorized into three groups: mild: <10% TILs, moderate: 10%-50% TILs, and severe: >50% TILs (Figures 2, 3).

Evaluation of iTILs was performed by identifying lymphocytes in direct contact with tumor cells. A scoring system, as described by Khoury et al. [4], was employed to assign an iTIL score for each case. For intratumoral lymphocytes (iTu-Ly), a semi-quantitative H-score method was used, which considered both the intensity of lymphocytic infiltration (graded from 0 to 3) and the proportion of the tumor displaying each grade. The grading criteria were as follows (refer to Figures 2, 3): Grade 0: no discernible lymphocytes; Grade 1: scattered lymphocytes detectable only at high magnification (40×); Grade 2: clearly visible lymphocytes at intermediate magnification (20×); Grade 3: dense infiltration obscuring tumor structures. The overall H-score was determined by multiplying each grade by the percentage of the tumor area it occupied and summing the results, yielding a possible range from 0 to 300.

**Figure 4:**
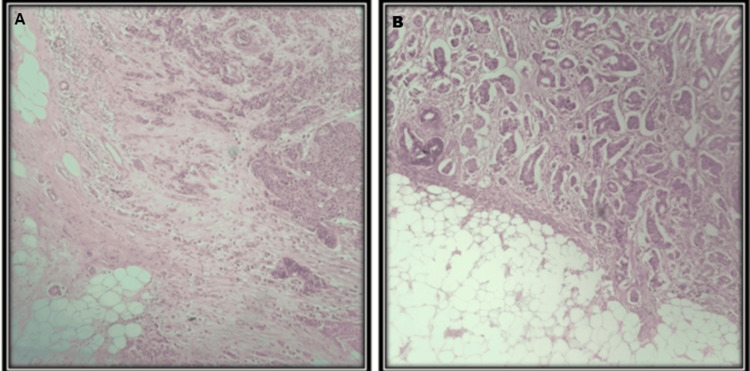
H&E-stained sections showing tumor budding in breast carcinoma (A) Multiple isolated single cells or small clusters (≤5 cells) at the invasive tumor front (200× magnification); (B) one to two tumor buds identified per high-power field, assessed according to the International Tumor Budding Consensus Conference (ITBCC) guidelines (200×). H&E: hematoxylin and eosin

Tumor buds were identified and quantified using a standardized approach. Initially, the invasive front of breast carcinoma was observed under low power (10× magnification) to locate tumor buds (Figure 2). A tumor bud was defined as an isolated single cancer cell or a cluster of up to five cancer cells. The highest bud count per field was recorded, excluding areas of necrosis or mucin. Further evaluation was performed under high-power field (40x magnification). Tumor buds were categorized into two groups based on density: low-grade and high-grade tumor buds (Figure 4).

Statistical analysis

The chi-squared test was used to examine the association between TB and clinicopathological characteristics. Statistical significance was set at p < 0.05.

## Results

Clinicopathological characteristics

The clinicopathological features of the 90 patients with invasive breast carcinoma are summarized in Table 1. The majority of tumors (63/90, 69.93%) were ≤5 cm in size. Most tumors were classified as Grade II (45/90, 49.95%) or Grade III (33/90, 36.63%). Lymph node metastasis was present in 50/90 cases (55.5%). A minority of patients (19/90, 21.11%) had received prior radiotherapy or chemotherapy.

**Table 1:** Distribution of the clinicopathological parameters of breast cancer Data represented as number of cases (N) and percentage (%). No statistical test was applied in this summary table. TNM: tumor size, lymph node involvement, and metastasis; sTILs: stromal tumor-infiltrating lymphocytes; iTILs: intratumoral tumor-infiltrating lymphocytes

Parameter	Category	No. of cases	Percentage
Laterality	Right	42	46.67
	Left	48	53.28
Age (in years)	<50	44	48.84
	>50	46	51.06
Tumor grade	Grade 1	11 (1 metaplastic)	12.21
	Grade 2	45	49.95
	Grade 3	33	36.63
Mitotic count	<5	33	36.63
	06-Oct	46	51.06
	>10	10 (1 metaplastic)	11.1
Tumor size (cm)	<5	63	69.93
	>5	27	29.97
Lymph node metastasis	Present	50	55.5
	Absent	39 (1 case no ln)	43.29
Lymphovascular invasion	Present	42	46.66
	Absent	48	53.33
TNM stage	T1	11	12.22
	T2	50	55.55
	T3	20	22.22
	T4	9	10
Percentage of sTILs	<10%	4	4.44
	10%-50%	54	60
	>50%	32	35.55
H score of iTILs	0-100	45	50
	101-200	43	47.77
	201-300	2	2.22
Tumor budding	High grade	54	60
	Low grade	36	40
Treatment status	No treatment	71	78.88
	Prior treatment	19	21.11

TB characteristics

High-grade TB was identified in 54 out of 90 patients (60.0%), while low-grade TB was present in 36 out of 90 patients (40.0%). Tables 2, 3 show the distribution of TB grades across different clinicopathological characteristics. Among patients below 50 years of age, 27 out of 44 (61.4%) had high-grade TB, while 17 out of 44 (38.6%) had low-grade TB. Among patients with tumor sizes < 5 cm, 31 out of 63 (49.2%) had high-grade TB, while 32 out of 63 (50.8%) had low-grade TB. In contrast, among those with tumor size ≥ 5 cm, 23 out of 27 (85.2%) had high-grade TB, while four out of 27 (14.8%) had low-grade TB. A significant association was observed between high-grade TB and lymph node metastasis. Among patients with high-grade TB, 35 out of 53 (66.0%) had lymph node metastasis. A significant proportion of patients with Grade II and Grade III tumors-50 out of 78 (64.1%)-also had high-grade TB. The majority of patients-68 out of 90 (75.6%)-were classified as Stage 2 or Stage 3, with a higher proportion of high-grade TB observed in these stages.

**Table 2:** This table highlights the association of tumor budding grade with age, tumor grade, and size Data are presented as the number of cases (N) for each category under low-grade and high-grade tumor budding. Statistical analysis was conducted using the chi-squared test, and the corresponding chi-squared values and p-values are shown for each variable. A p-value of <0.05 was considered statistically significant.

Parameter	Category	Low-grade budding (N)	High-grade budding (N)	Chi-squared value	p-value
Age	<50 years	17	27	0.07	0.799
	≥50 years	19	27		
Tumor grade	Grade I	8	3	9.41	0.009
	Grade II	19	26		
	Grade III	9	24		
Tumor size	<5 cm	32	31	5.07	0.024
	≥5 cm	4	23		

**Table 3:** This table highlights the association of tumor budding grade with lymph node status, mitotic count, lymphovascular invasion, and TNM stage Data are presented as the number of cases (N) for each category under low-grade and high-grade tumor budding. Statistical analysis was conducted using the chi-squared test, and the corresponding chi-squared values and p-values are shown for each variable. A p-value of <0.05 was considered statistically significant. TNM: tumor size, lymph node involvement, and metastasis

Parameter	Category	Low-grade budding (N)	High-grade budding (N)	Chi-squared value	p-value
Lymph node metastasis	Present	15	35	5.16	0.023
	Absent	21	18		
Mitotic count	<5	18	15	5.11	0.078
	06-Oct	15	31		
	>10	3	7		
Lymphovascular invasion	Present	10	32	8.64	0.003
	Absent	26	22		
TNM stage	T1	10	1	13.36	0.004
	T2	19	31		
	T3	4	16		
	T4	3	6		

TIL analysis

The mean values of sTILs and iTILs were calculated as a percentage and a score out of 300, respectively. The results are presented in Table 1. Out of 90 cases, 32 patients (35.55%) had sTILs greater than 50%. The majority of patients-45 out of 90 (50.0%)-had an iTIL score between 0 and 101, while only two out of 90 patients (2.22%) had a score above 200. The mean values of sTILs and iTILs exhibited a decreasing trend with increasing tumor grade, size, and mitotic activity. This correlation was statistically significant for tumor grade and mitosis (p = 0.000 and p = 0.002 for sTILs; p = 0.000 and p = 0.000 for iTILs). In terms of TNM (tumor size, lymph node involvement, and metastasis) staging, the p-value was significant for iTILs (p = 0.052) (Table 3). The mean values of sTILs and iTILs were higher in cases with lower tumor grade and smaller tumor size. This correlation was statistically significant for tumor grade (p = 0.000 for both sTILs and iTILs). A progressive decline in the mean values of both sTILs and iTILs was observed with advancing TNM stage, from Stage I to Stage IV. This trend reached statistical significance for iTILs. Table 4 summarizes the mean sTIL and iTIL values across different clinicopathological variables.

**Table 4:** Mean values of sTIL and iTIL in the context of various clinicopathological parameters. Statistical comparisons were performed for each variable sTIL: stromal tumor-infiltrating lymphocyte; iTIL: intratumoral tumor-infiltrating lymphocyte; TNM: tumor size, lymph node involvement, and metastasis

Parameters (n = 90)	Categories	No. of cases	sTILs (mean ± SD)	p-value	iTILs (mean ± SD)	p-value
Age (years)	<50	44	44.72 ± 26.90	0.277	104.88 ± 53.18	0.993
	>50	46	36.04 ± 23.84		103.15 ± 38.38	
Tumor grade	Grade I	11	67.27 ± 21.25	0	190 ± 18.21	0
	Grade II	45	45.24 ± 25.64		111.88 ± 25.50	
	Grade III	33	24.54 ± 15.39		63.78 ± 25.34	
Tumor size (in cm)	<5	63	40.44 ± 25.26	0.966	105.55 ± 43.99	0.193
	>5	27	39.92 ± 26.84		100.37 ± 50.86	
Lymph node metastasis (n = 89)	Present	50	41.36 ± 27.00	0.408	103.1 ± 49.17	0.474
	Absent	39	49.05 ± 24.31		105.12 ± 42.76	
Mitotic count (/10 hpf) (n = 90)	<5	33	51.96 ± 25.87	0.002	138.48 ± 43.85	0
	06-Oct	46	35.45 ± 24.61		89.45 ± 33.56	
	>10	10	24.00 ± 12.20		54.5 ± 16.94	
Lymphovascular invasion (n = 90)	Present	42	37.64 ± 25.41	0.494	97.02 ± 42.03	0.72
	Absent	48	42.60 ± 25.81		110.10 ± 48.75	
TNM stage (n = 90)	T1	11	55.45 ± 27.09	0.232	135 ± 51.65	0.052
	T2	50	38.76 ± 23.53		101.9 ± 39.55	
	T3	20	41.15 ± 27.12		97.75 ± 56.29	
	T4	9	28.33 ± 24.15		91.66 ± 30.55	

Correlation analysis

To assess the relationship between TILs and various clinicopathological features in breast carcinoma, Pearson correlation coefficients were computed. sTILs demonstrated significant inverse correlations with tumor grade (r = -0.505), tumor size (r = 0.005), TNM stage (r = -0.127), and mitotic count (r = -0.330). Likewise, iTILs showed significant negative correlations with tumor grade (r = -0.672), mitotic activity (r = -0.517), and TNM stage (r = -0.205), while showing a weak inverse correlation with tumor size (r = -0.139). A strong positive correlation was observed between sTILs and iTILs (r = 0.426), suggesting a parallel immune response in both stromal and intratumoral compartments.

TIL distribution across differentiation degrees

Table 5 shows the distribution of TILs across different degrees of tumor differentiation. Out of 89 samples, 53 (59.55%) had low TILs, and 36 (40.44%) had high TILs. The distribution of TILs varied significantly across tumor differentiation grades. Among well-differentiated tumors (Grade 1, n = 11), two cases (18.18%) had low TILs, while nine cases (81.81%) had high TILs. In moderately differentiated tumors (Grade 2, n = 45), 20 cases (44.44%) had low TILs and 25 cases (55.55%) had high TILs. Among poorly differentiated tumors (Grade 3, n = 33), 31 cases (93.93%) had low TILs and only two cases (6.06%) had high TILs.

**Table 5:** Degree of TILs by histopathological grade Data represented as the number of cases (N). TIL distribution (<50% vs. >50%) was compared across histopathological grades. Statistical analysis was done using the chi-squared test. A p-value < 0.05 was considered significant. TIL: tumor-infiltrating lymphocyte; sTIL: stromal tumor-infiltrating lymphocyte

	Histopathological grade (differentiation)			Total
Degree of sTILs	Grade 1	Grade 2	Grade 3	
Low (<50%)	2	20	31	53
High (>50%)	9	25	2	36
Total	11	45	33	89

Our study investigated the correlation between sTILs and TB, as presented in Table 6. To analyze this relationship, sTILs were categorized into three groups: Group 1 (<10% sTILs, n = 4), Group 2 (10%-50% sTILs, n = 54), and Group 3 (>50% sTILs, n = 32). Among cases with <10% sTILs, one out of four (25.0%) had low-grade TB, and three out of four (75.0%) had high-grade TB. In the 10%-50% sTIL group, 17 out of 54 cases (31.5%) had low-grade TB, while 37 (68.5%) had high-grade TB. In the >50% sTIL group, 18 out of 32 cases (56.25%) had low-grade TB and 14 (43.75%) had high-grade TB. The chi-squared test revealed a p-value of 0.124, and the Pearson correlation coefficient was 0.163. Although the p-value did not reach statistical significance (typically considered as p < 0.05), the correlation coefficient suggests a weak-to-moderate positive association between sTILs and TB. This indicates that higher levels of sTILs may be associated with increased TB, though the finding was not statistically significant.

**Table 6:** Correlation between sTILs and TB Data represented as the number of cases (N). Chi-squared test used to compare sTIL categories across tumor budding (TB) grades. Pearson's correlation coefficient used to assess strength of association. A p-value < 0.05 was considered statistically significant. sTIL: stromal tumor-infiltrating lymphocyte

	Low-grade budding	High-grade budding	Total	p-value	Pearson correlation
sTILs <10%	1	3	4		
sTILs 10%-50%	17	37	54	0.124	0.163
sTILs >50%	18	14	32		
Total	36 (40%)	54 (60%)	90		

## Discussion

This study explored the prognostic relevance of TILs and TB in invasive breast carcinoma. Accumulating evidence highlights that the presence of immune cells within tumors and the surrounding stroma significantly influences clinical outcomes in breast and other solid malignancies [6,7]. B and T lymphocytes are key mediators of adaptive immunity, contributing to long-lasting and robust anti-tumor responses [8-10]. TILs are now considered predictive not only of therapeutic response-particularly to immunotherapy and chemotherapy-but also serve as important prognostic indicators [11]. Their presence reflects a dynamic interaction between tumor biology and host immunity within the tumor microenvironment, which influences treatment outcomes, including responses to immune checkpoint inhibitors.

TB has been well documented as a negative prognostic factor, especially in colorectal cancer [6], and is increasingly being investigated in breast carcinoma. It is thought to reflect the EMT, a biological process that promotes invasiveness and metastatic potential. TB, alongside other markers like lymph node involvement, receptor status, and Bloom-Richardson (BR) score, offers additional insight into tumor behavior and prognosis. As a driver of tumor progression, TB is considered a marker of poor clinical outcome.

The current study supports previously reported findings in breast carcinoma. A higher prevalence of cases was observed in patients aged above 50, echoing observations from Buch et al. [12], who linked advanced age with less favorable prognoses. We applied a tumor bud counting method similar to that of Patel and Gupta [13], using a 10× objective lens across 10 fields, allowing broader visualization at lower magnification. Conversely, other studies, such as those by Abd El Khalek and Halim [14] and Agarwal et al. [15], used 20× objectives. While our study utilized only H&E-stained sections (as in Singh et al. [16]), other researchers, including Masilamani and Kanmani and Mozarowski et al. [17,18], incorporated immunohistochemistry to improve the accuracy of bud detection.

Our results revealed a statistically significant association between high TB and several adverse clinicopathological factors-including larger tumor size, higher grade, lymph node metastasis, and advanced stage-findings that align with studies by Rathod et al. [19] and Salhia et al. [20]. The observed link between TB and tumor progression (p = 0.024) mirrors similar associations in earlier studies [16,20]. Although Okcu et al. [21] reported that tumor size had a stronger correlation with high-grade TB than tumor grade, our findings show a significant relationship with both.

While the ITILWG recommends focusing on sTILs [8,9], variations in TIL counts across tumor compartments have been documented. Both stromal lymphocytes and iTu-Ly contribute to anti-tumor immunity and modulate the immune landscape of the tumor microenvironment. Our analysis included both sTILs and iTu-Ly, adopting an approach similar to Iseki et al.'s study [22], and showed a marked variation in TIL levels across differentiation grades. Higher TIL scores were associated with well-differentiated tumors, whereas poorly differentiated tumors demonstrated lower TIL infiltration. This is consistent with the role of T lymphocytes-particularly CD8+ T cells-in suppressing tumor growth via cytokine production, such as IL-2 and IL-15, and direct cytotoxic activity [23].

Angelico et al. [24] also noted that advanced-stage tumors were associated with low TIL counts (<10%). In our cohort, most Grade 3 tumors had sTILs below 50%, further supporting the idea that aggressive tumor phenotypes may suppress host immune responses. A decline in sTIL levels was also seen in cases with lymph node metastasis, implying that inadequate immune infiltration may facilitate dissemination. Although iTILs are more challenging to quantify on H&E slides and are often sparse, both sTIL and iTIL are predictive of neoadjuvant chemotherapy response. This is also supported by our finding of a strong correlation (r = 0.426) between the two.

As described by Pujani et al. [1], tumor size and axillary lymph node involvement remain among the most critical independent prognostic factors in breast cancer. While our study did not find statistically significant associations between TILs and these parameters, we observed a favorable trend toward higher TILs in cases with smaller tumors and no nodal metastasis. This discrepancy may stem from the inclusion of patients with prior neoadjuvant chemotherapy, which can modulate immune infiltration.

Furthermore, our analysis indicated a positive correlation between TIL levels and LVI, in line with earlier reports [20,25-27]. This might reflect the role of immune cells in secreting angiogenic factors like VEGF and FGF, which promote neovascularization and enhance tumor spread via lymphatics.

We also assessed the relationship between TILs and TB. Notably, cases with <50% sTIL infiltration were more likely to show high-grade TB, while those with >50% sTILs were commonly associated with low-grade TB. These findings suggest that TILs and TB may function as counterbalancing elements within the tumor microenvironment, where a robust immune presence could limit tumor dissemination.

A key strength of this study is that it is among the few from our region to systematically evaluate both TILs and TB in invasive breast carcinoma using standardized international guidelines. The clear objectives and uniform methodology add to the reproducibility of findings. However, our study has some limitations, including a relatively small sample size, the lack of long-term clinical follow-up, and potential interobserver variability in scoring TILs and TB. Future studies with larger, prospective cohorts and survival analysis are warranted to validate these observations.

## Conclusions

Our study highlights the significant associations of TILs and TB with key clinicopathological features in invasive breast carcinoma. High TB correlated with aggressive characteristics such as higher tumor grade, lymph node metastasis, and advanced stage, whereas higher levels of sTILs and iTILs were linked with better differentiation, reduced mitotic activity, and absence of nodal involvement. The observed inverse correlation between TILs and TB suggests a possible interplay between immune-mediated tumor suppression and tumor progression.

While these findings reinforce the potential utility of TILs and TB in refining risk assessment, the absence of survival data limits direct prognostic interpretation. Therefore, larger prospective studies with long-term follow-up are warranted to validate the prognostic significance of these markers and explore their role in clinical decision-making.
